# Deletion of the Loop Linking Two Domains of Exo-Inulinase InuAMN8 Diminished the Enzymatic Thermo-Halo-Alcohol Tolerance

**DOI:** 10.3389/fmicb.2022.924447

**Published:** 2022-06-23

**Authors:** Xiaolong Cen, Rui Zhang, Limei He, Xianghua Tang, Qian Wu, Junpei Zhou, Zunxi Huang

**Affiliations:** ^1^Engineering Research Center of Sustainable Development and Utilization of Biomass Energy, Ministry of Education, Yunnan Normal University, Kunming, China; ^2^College of Life Sciences, Yunnan Normal University, Kunming, China; ^3^Key Laboratory of Biomass Energy and Environmental Biotechnology, Yunnan Normal University, Kunming, China; ^4^Key Laboratory of Yunnan Provincial Education Department for Plateau Characteristic Food Enzymes, Yunnan Normal University, Kunming, China

**Keywords:** inulinase, loop, thermostability, salt, alcohol, structure, mechanism, mutagenesis

## Abstract

Inulin is the rich water-soluble storage polysaccharide after starch in nature, and utilization of inulin through hydrolysis of exo-inulinases has attracted much attention. Thermo-halo-alcohol tolerance is essential for exo-inulinase applications, while no report reveals the molecular basis involved in halo-alcohol tolerance of exo-inulinases *via* experimental data. In this study, two loops of exo-inulinase InuAMN8, including the loop built with ^360^GHVRLGPQP^368^ linking domains of Glyco_hydro_32N and Glyco_hydro_32C and another loop built with ^169^GGAG^172^ in the catalytic domain, were deleted to generate mutants MutG360Δ9 and MutG169Δ4, respectively. After heterologous expression, purification, and dialysis, InuAMN8, MutG169Δ4, and MutG360Δ9 showed half-lives of 144, 151, and 7 min at 50°C, respectively. InuAMN8 and MutG169Δ4 were very stable, while MutG360Δ9 showed a half-life of approximately 60 min in 5.0% (w/v) NaCl, and they showed half-lives of approximately 60 min in 25.0, 25.0, and 5.0% (w/v) ethanol, respectively. Structural analysis indicated that two cation-π bonds, which contributed to thermal properties of InuAMN8 at high temperatures, broke in MutG360Δ9. Four basic amino acid residues were exposed to the structural surface of MutG360Δ9 and formed positive and neutral electrostatic potential that caused detrimental effects on halo-alcohol tolerance. The study may provide a better understanding of the loop-function relationships that are involved in thermo-halo-alcohol adaptation of enzymes in extreme environment.

## Introduction

Jerusalem artichoke is widely cultivated in China, even in saline-alkaline soils or coastal shoals, as a non-grain crop and also widely distributed in tropical and temperate countries across the world, since the crop has the advantages of rapid growth as well as the high resistance to pets, cold, drought, salt, and alkali conditions (Qiu et al., 2018; Singh et al., 2019). Tuberous roots of Jerusalem artichoke store inulin with an abundant content as high as 80% of the carbohydrates (Qiu et al., 2018). Inulin is well-known as the rich storage polysaccharide after starch in nature, thus, its utilization has attracted much attention (Singh et al., 2019).

Inulin is a hot-water-soluble polyfructan with a linear structure linked by β-D-(2**→**1) fructosides and ended by a (1**→**2) α-D-glucose unit (Qiu et al., 2018; Singh et al., 2019). Compared with other renewable biomass such as lignin, cellulose, hemicellulose, and chitin, biotransformation of inulin is easier on account of the higher water solubility and lower structure heterogeneity and complexity (Saha, 2003; Geib et al., 2008; Stoykov et al., 2015; Singh et al., 2019).

Hydrolysis of inulin using exo-inulinases is a convenient and efficacious biotransformation way owing to that a high-content (90–95%) fructose syrup, which is more than 2-fold fructose concentration produced *via* conventional multienzymatic transformation of starch, is directly produced through the single-step hydrolysis process (Singh et al., 2018a). Fructose syrup is a generally recognized as safe (GRAS) food ingredient and has been widely used as a sweetener in various food and beverage such as Coca-Cola and Pepsi (Singh et al., 2018a). Fructose is also being used in pharmaceutical industries as the capsule formulation and infusion or injection solution and used as fermentable sugar to produce various valuable chemicals such as butanol, ethanol, single-cell oils, sorbitol, lactic acid, succinic acid, and poly-(γ-glutamic acid) (Qiu et al., 2018; Singh et al., 2019, 2022).

Thermo-halo-alcohol tolerance is essential for enzyme applications in various biotechnology industries. For example, enzymes with good thermal tolerance usually show the advantages of a high reaction rate and solubility of substrates at a high temperature (Xu et al., 2020); salt-tolerant enzymes usually show the ability of processing high salt and marine food such as marine algae, pickles, and sauces, yielding biochemicals and biofuel using sea water (Warden et al., 2015; Cao et al., 2021); alcohol-tolerant enzymes suit for simultaneous saccharification and fermentation using microorganisms to transform biomass to butanol, ethanol, acetone, and other useful chemicals (Li et al., 2018; Singh et al., 2022).

Exo-inulinases are classified in family 32 of glycoside hydrolases (GH32), which usually consist of two domains, with the N-terminal catalytic domain belonging to Glyco_hydro_32N (PF00251) and the C-terminal domain belonging to Glyco_hydro_32C (PF08244) (Mistry et al., 2021). To date, studies on thermal properties and substrate recognition, as well as related mechanisms of exo-inulinases, have been reported (Nagem et al., 2004; Arjomand et al., 2016; Zhou S. H. et al., 2016; Singh et al., 2018b; Germec and Turhan, 2019, 2020; Ma et al., 2019, 2020; He et al., 2020, 2022; Zhang et al., 2020; Wang et al., 2021). Among these studies, deletion of loops at the N-terminal tail and catalytic domain influences the thermal performance of exo-inulinases (Arjomand et al., 2016; He et al., 2020, 2022). However, the effects of loops, especially the loop in the linking region, on the thermo-halo-alcohol tolerance of exo-inulinases remain unclear.

Previously, the low-temperature-active and salt-tolerant exo-inulinase InuAMN8 was isolated in our lab from *Arthrobacter* sp. MN8, which was a cold-adapted bacterium harbored in lead-zinc-rich soil (Zhou et al., 2015a). To the best of our knowledge, only InuAMN8 shows an optimal exo-inulinase activity at 35°C (Zhou et al., 2015a), while others show optimal exo-inulinase activity at the temperature range of equal to or higher than 40°C (Kango and Jain, 2011; Singh et al., 2017). In this study, the loop linking domains of Glyco_hydro_32N and Glyco_hydro_32C of InuAMN8 were deleted, and the effects of the loop deletion on the thermo-halo-alcohol tolerance and structural properties were investigated. The study may provide a better understanding of the loop-function relationships that are involved in thermo-halo-alcohol adaptation of enzymes in extreme environment.

## Materials and Methods

### Chemicals, Plasmids, and Strains

The commercial reagents include Mut Express II Fast Mutagenesis Kit for enzyme mutagenesis (Vazyme Biotech, Nanjing, China), isopropyl-β-D-1-thiogalactopyranoside for recombinant enzyme induction (Amresco, Solon, OH, United States), *Escherichia coli* BL21 (DE3) for recombinant enzyme expression (TransGen, Beijing, China), nickel-NTA agarose for recombinant enzyme purification (Qiagen, Valencia, CA, United States), dialysis bag with a molecular weight cutoff of 44 kDa for removing the elution reagent of enzyme purification (Biosharp, Hefei, China), substrate inulin for enzymatic reaction (Thermo Fisher Scientific, Waltham, MA, United States), microplate for enzymatic assay (Corning, NY, United States), and silica gel G plate for thin-layer chromatography (Haiyang, Qingdao, China). Other commercial reagents are of analytical grade and purchased from regular suppliers.

Previously, exo-inulinase InuAMN8 (accession number AGC01505) was isolated from *Arthrobacter* sp. MN8 deposited in the Strains Collection of the Yunnan Institute of Microbiology under registration no. YMF 4.00006, and InuAMN8-encoding gene (accession number JQ863111) was ligated to vector *pEASY*-E1 (TransGen) and heterologously expressed in *E. coli* BL21 (DE3) (Zhou et al., 2015a).

### Multiple Amino Acid Sequences Alignment and Structure Modeling

Representative amino acid sequences of GH32 from the Pfam database (Mistry et al., 2021) and InuAMN8 were aligned using Clustal X (Chenna et al., 2003), then manually adjusted. The tertiary structures of InuAMN8 and its mutants were homologously modeled using SwissModel (Guex et al., 2009), and model quality was evaluated using Verify3D (Eisenberg et al., 1997) and PROCHECK (Laskowski et al., 1993) programs ran on the SAVES server of the UCLA-DOE Institute for Genomics and Proteomics. Structures of InuAMN8 and its mutants were visualized using the Discovery Studio software (Accelrys, San Diego, CA, United States).

### Vectors of Mutants Construction

Primer sets for mutagenesis were designed using the CE Design software (Vazyme Biotech), with the expression plasmid (*pEASY-inuAMN8*) of wild-type InuAMN8 as a sequence template, which was constructed previously (Zhou et al., 2015a). The primer set for mutant MutG169Δ4 (deletion of residues ^169^GGAG^172^) was TGGTACGACAGTTACTGGGTGATGGTCGCCGTC and CCA GTAACTGTCGTACCAAAAAACCTTTGGATC. The primer set for mutant MutG360Δ9 (deletion of residues ^360^GHVRLGPQP^368^) was AGCGGGAAACATTGGCGTCCGGC GTTCTG and ACGCCAATGTTTCCCGCTCCGGCAA. The primer set for mutant MutV376Δ5 (deletion of residues ^376^VPAAA^380^) was GTTCTGGACTCCGTGGCGCGGATCGAC and CGCCACGGAGTCCAGAACGCCGGACGC.

According to the manufacturer’s instructions of Mut Express II Fast Mutagenesis Kit V2, mutated vectors were obtained *via* amplification with polymerase chain reaction, digestion with restriction enzyme *Dpn*I, and homologous recombination with the enzyme Exnase.

### Recombinant Enzyme Induction and Heterologous Expression

The *E. coli* BL21 (DE3) competent cells were CaCl_2_-heat-shocked for the transformation of the mutated vectors. After that, mutated vectors were methylated in *E. coli* BL21 (DE3) cells. Positive transformants harboring the mutated sequences were individually confirmed by DNA sequencing of plasmids (Tsingke, Beijing, China).

Details of mutated enzyme induction and heterologous expression are the same as that of wild-type InuAMN8 and have been described previously (Zhou et al., 2015a). Briefly, the induction and expression of recombinant enzyme were performed using isopropyl-β-D-1-thiogalactopyranoside as an induction reagent, Luria-Bertani broth with 100 mg ml^–1^ ampicillin as culture medium, and shaking at 200 rpm and 20°C for approximately 20 h as induction conditions.

### Recombinant Enzyme Purification and Dialysis

Wild-type InuAMN8 and its mutants were expressed inside the cells of *E. coli* BL21 (DE3). The host cells were harvested by centrifugation at 12,000 × *g* for 10 min at 20°C and then disrupted by sonication (20–24 kHz) on ice, as described previously (Zhou et al., 2015a). Recombinant enzymes in the sonication-disrupted solution were purified using immobilized His_6_-tag affinity chromatography, with the purification reagents containing 20 mM Tris–HCl (pH 7.2), 0.5 M NaCl, 10% (w/v) glycerol, and 300 mM imidazole.

With regard to the effects of NaCl and glycerol on exo-inulinase property (Zhou et al., 2014, 2015a), the elution of affinity chromatography, which contained purified enzymes, were dialyzed with the dialysis bag against McIlvaine buffer (pH 7.0) at 12°C, shaking at 60 rpm for an appropriate time.

The purity of purified wild-type InuAMN8 and its mutants were evaluated by sodium dodecyl sulfate-polyacrylamide gel electrophoresis (SDS-PAGE) experiments.

### Recombinant Enzyme Characterization

Exo-inulinase activity of purified wild-type InuAMN8 and its mutants were determined by the classic 3,5-dinitrosalicylic acid (DNS) method (Miller, 1959), with 450 μl of 0.5% (w/v) inulin solution used as the substrate. After the substrate preheat at the reaction temperature, 50 μl of purified wild-type InuAMN8 or the mutated enzyme was pipetted into the substrate solution to initiate the catalytic activity. To stop the catalytic activity, 750 μl of the DNS reagent was pipetted into the reaction mixture. The hydrolytic products, including fructose and a small amount of glucose, reacted with DNS to show a reddish-brown product in a boiling water bath. The absorption of the reddish-brown product was measured at 540 nm using a microplate reader. One unit of exo-inulinase activity was defined as the amount of enzyme releasing 1 μmol of fructose per minute. Experiments of enzyme characterization were performed in triplicate.

Activity determination of purified wild-type InuAMN8 and its mutants was individually carried out in pH 7.0 McIlvaine buffer at 0–60°C, 5.0–25.0% (w/v) NaCl, or 3.0–25.0% (v/v) ethanol. Stability determination was to measure the residual activity at 37°C in pH 7.0 McIlvaine buffer after individually incubating these purified enzymes at 50°C for 10–60 min, 5.0–25.0% (w/v) NaCl for 60 min, or 3.0–25.0% (v/v) ethanol for 60 min in the absence of inulin. More details have been described in the previous study (Zhou et al., 2015a). Half-lives of enzymes at 50°C (*t*_1/2_) were calculated according to the stability data using the equation: Half-life = ln0.5/(–*k*_*d*_), where *k*_*d*_ is the slope plotted with ln(activity) vs. time.

The thin-layer chromatography method, performed as described previously (Zhou et al., 2015a), was employed to visualize the hydrolysis products of purified wild-type InuAMN8 and its mutants toward inulin, after enzymatic reactions carried out at 37°C, pH 7.0 for 4 h.

### Structural Analyses of Enzymes

Intraprotein interactions of InuAMN8 and its mutants, including salt bridges (oxygen-nitrogen distance cutoff: 3.2Å) and energetically significant cation-π interactions (distance cutoff: 6.0 Å), were predicted using VMD (Humphrey et al., 1996) and CaPTURE (Gallivan and Dougherty, 1999), respectively, and visualized using the Discovery Studio software (Accelrys).

## Results

### Multiple Amino Acid Sequences Alignment and Structure Modeling

The homology model of InuAMN8 ranking first in the SwissModel modeling results was selected for the study. The model used the exo-inulinase from *Aspergillus awamori* var. 2250 (PDB ID 1Y4W) as template (Arand et al., 2002), with an amino acid sequence identity of 42.1%. The Ramachandran plot of the InuAMN8 model generated with PROCHECK showed 99% residues in allowed regions (Supplementary Figure 1). VERIFY3D results of the InuAMN8 model indicated that 99.39% of the residues had an averaged 3D-1D score equal to or higher than 0.2 (Supplementary Figure 2).

**FIGURE 1:**
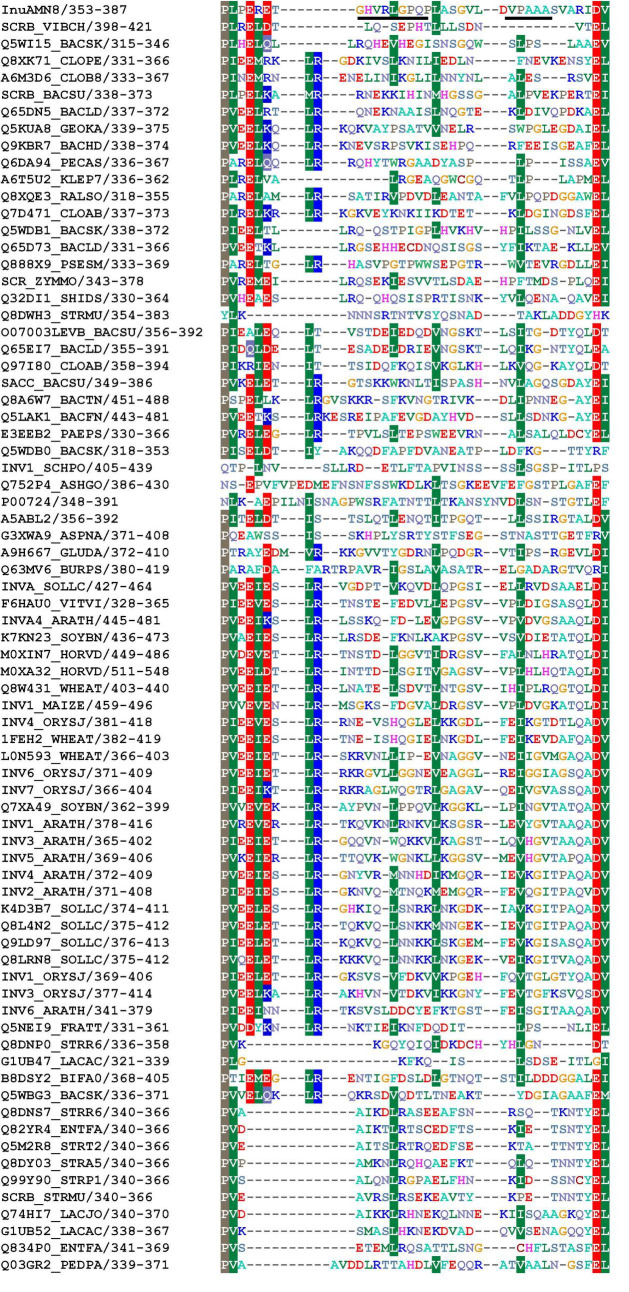
Partial alignment of amino acid residues of InuAMN8 with seed sequences of GH 32 from Pfam database (Mistry et al., 2021). Residues ^360^GHVRLGPQP^368^ and ^376^VPAAA^380^ selected for mutagenesis are underlined.

**FIGURE 2:**
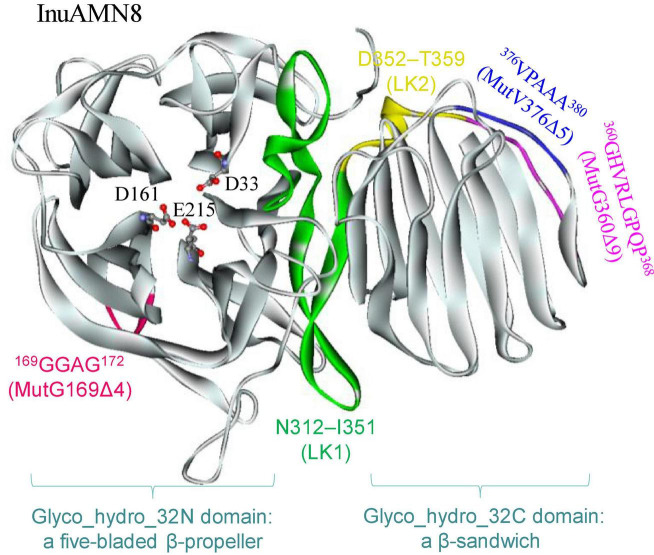
The tertiary structure of InuAMN8. Catalytic amino acid residues are shown in ball and stick form.

To identify the suitable mutagenesis region, multiple amino acid sequences alignment and structure model were combined to analyze. The alignment of InuAMN8 with representative amino acid sequences of GH32 showed an unconserved region from residues P355–I385 of InuAMN8 (Figure 1). The InuAMN8 model indicated that two loops were built with residues P355–I385, including one loop built with ^360^GHVRLGPQP^368^ linking domains of Glyco_hydro_32N and Glyco_hydro_32C, as well as another loop built with ^376^VPAAA^380^ (Figure 2). Thus, the deletion of ^360^GHVRLGPQP^368^ and ^376^VPAAA^380^ was performed to generate mutants MutG360Δ9 and MutV376Δ5, respectively. Furthermore, domains of Glyco_hydro_32N and Glyco_hydro_32C of InuAMN8 were also linked by a 3_10_-helix plus β-strands structure built with residues N312–I351 (designated as LK1) and a 3_10_-helix structure built with residues D352–T359 (designated as LK2) (Figure 2).

Nine Ω-loops were observed in the catalytic domain of Glyco_hydro_32N of exo-inulinases (Arjomand et al., 2016). Two of the nine Ω-loops have been deleted previously and resulted in thermostability loss of exo-inulinases (Arjomand et al., 2016; He et al., 2022). To compare the effects between the deletion of the Ω-loop in the catalytic domain and the loop built with ^360^GHVRLGPQP^368^ in the linking region on thermo-halo-alcohol tolerance, the Ω-loop built with ^169^GGAG^172^ in the catalytic domain was also deleted to generate the mutant MutG169Δ4 (Figure 2).

### Enzyme Expression in *Escherichia coli*

The expression vectors for mutants MutG169Δ4, MutG360Δ9, and MutV376Δ5 were successfully constructed using the Mut Express II Fast Mutagenesis Kit V2 and then transformed to *E. coli* BL21 (DE3) competent cells separately. After induction of positive transformants with isopropyl-β-D-1-thiogalactopyranoside, enzymatic activities of MutG169Δ4 and MutG360Δ9 toward inulin were observed in the supernatant of disrupted cells solution. In contrast, the enzymatic activity of MutV376Δ5 was not observed.

**FIGURE 3:**
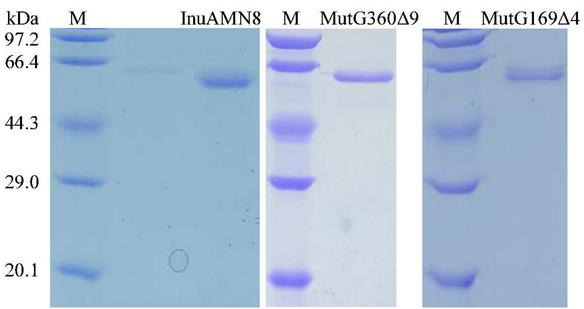
Sodium dodecyl sulfate-polyacrylamide gel electrophoresis analysis of purified wild-type InuAMN8 and its mutants. Lane M, protein molecular weight marker.

Crude MutG169Δ4, MutG360Δ9, and MutV376Δ5, as well as the wild-type InuAMN8 in the supernatant of disrupted cells solution, were individually loaded onto nickel-NTA agarose gel columns and eluted with the purification reagents containing 20 mM Tris–HCl (pH 7.2), 0.5 M NaCl, 10% (w/v) glycerol, and 300 mM imidazole. Elutions were dialyzed against McIlvaine buffer (pH 7.0) on account of the effects of NaCl and glycerol in the elution on exo-inulinase property (Zhou et al., 2014, 2015a). As shown in Figure 3, SDS-PAGE analysis indicated that the wild-type InuAMN8 and its mutants MutG169Δ4 and MutG360Δ9 were successfully heterologously expressed and purified to electrophoretic purity, while the band of mutant MutV376Δ5 was not observed. SDS-PAGE and enzymatic activity assay indicated that the mutant MutV376Δ5 was not expressed in *E. coli* BL21 (DE3). Thus, thermo-halo-alcohol characteristics of MutV376Δ5 were not determined.

### Thermo-Halo-Alcohol Characteristics of Purified Enzymes

**FIGURE 4:**
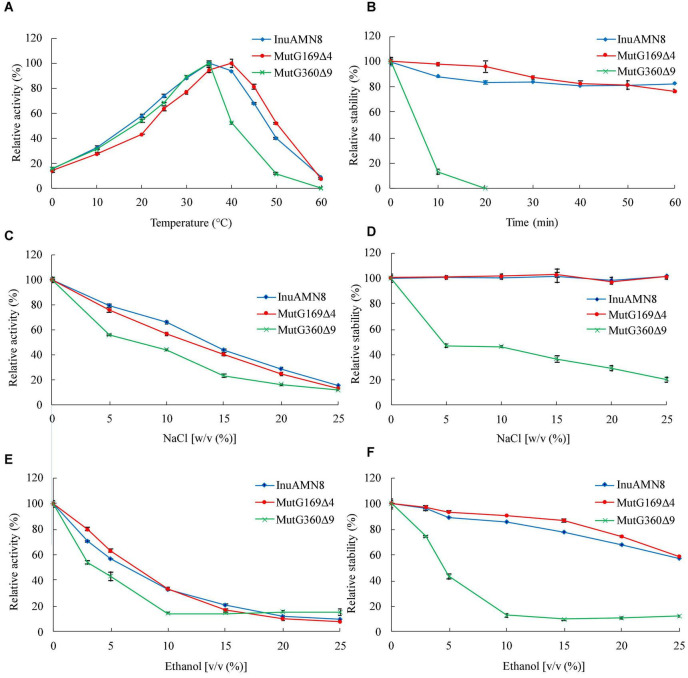
Thermo-halo-alcohol characteristics of purified wild-type InuAMN8 and its mutants. **(A,C,E)** indicate activity assay; **(B,D,F)** indicate stability assay. The error bars represent the means ± SD (*n* = 3).

The thermal activity assay indicated that purified wild-type InuAMN8 and its mutants MutG169Δ4 and MutG360Δ9 were maximally active at 35, 40, and 35°C, respectively (Figure 4A). All of the three enzymes showed approximately 15% relative activity at 0°C, while InuAMN8, MutG169Δ4, and MutG360Δ9 showed 40.0, 52.1, and 11.6% relative activities at 50°C, respectively (Figure 4A). The thermostability assay indicated that purified wild-type InuAMN8 showed 88.3–82.4% residual activities after incubation of the enzyme at 50°C for 10–60 min (Figure 4B). At the same incubation conditions, MutG169Δ4 showed 97.9–76.2% residual activities, while MutG360Δ9 showed only 12.9–0% residual activities (Figure 4B). Values of *t*_1/2_ at 50°C were 144, 151, and 7 min for InuAMN8, MutG169Δ4, and MutG360Δ9, respectively. Thus, the above results revealed that the thermal tolerance of exo-inulinase InuAMN8 was affected slightly after ^169^GGAG^172^ deletion while that diminished greatly after ^360^GHVRLGPQP^368^ deletion.

The halo tolerance and alcohol tolerance of exo-inulinase InuAMN8 were also affected slightly after ^169^GGAG^172^ deletion, while that were diminished greatly after ^360^GHVRLGPQP^368^ deletion. Adding 5.0–20.0% (w/v) NaCl to the reaction mixture, InuAMN8, MutG169Δ4, and MutG360Δ9 showed 79.1–28.7%, 75.7–24.6%, and 55.8–16.2% of the initial activity, respectively (Figure 4C). After incubation of purified enzymes in 5.0–25.0% (w/v) NaCl for 60 min, InuAMN8 and MutG169Δ4 were very stable as activity loss was not observed, while MutG360Δ9 showed only 47.0–20.5% residual activities, with a half-life value of approximately 60 min in 5.0% (w/v) NaCl (Figure 4D). Adding 3.0–10.0% (v/v) ethanol to the reaction mixture, InuAMN8, MutG169Δ4, and MutG360Δ9 showed 70.8–32.9, 79.6–33.6, and 53.8–14.6% of the initial activity, respectively (Figure 4E). After incubation of purified enzymes in 3.0–25.0% (w/v) ethanol for 60 min, InuAMN8, MutG169Δ4, and MutG360Δ9 showed 96.3–57.4, 97.0–58.7, and 74.3–12.2% residual activities, respectively (Figure 4F). Half-life values of InuAMN8, MutG169Δ4, and MutG360Δ9 were approximately 60 min in 25.0, 25.0, and 5.0% (w/v) ethanol, respectively.

Regarding the unclear mechanisms of exo-action and endo-action of GH 32 exo-inulinases, thin-layer chromatography was performed. It showed that fructose was the end-product of inulin hydrolysis by InuAMN8, MutG169Δ4, and MutG360Δ9. The results revealed that the deletion of ^169^GGAG^172^ and ^360^GHVRLGPQP^368^ did not change the exo-action mode of the enzyme.

### Structural Characteristics

To compare structural characteristics between wild-type InuAMN8 and its mutants, the homology models of MutG169Δ4 and MutG360Δ9 were also successfully built using the exo-inulinase from *A. awamori* var. 2250 (PDB ID 1Y4W) as a template. The Ramachandran plot of MutG169Δ4 and MutG360Δ9 models generated with PROCHECK showed 99.0 and 98.8% residues in allowed regions, respectively (Supplementary Figure 1). VERIFY3D results of MutG169Δ4 and MutG360Δ9 models indicated that 99.8 and 92.7% of the residues had an averaged 3D-1D score equal to or higher than 0.2, respectively (Supplementary Figure 2).

**FIGURE 5:**
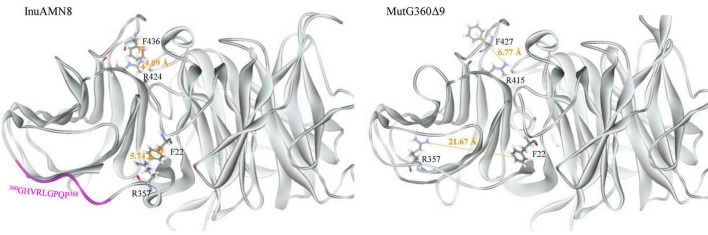
Comparisons of cation-π interactions between wild-type InuAMN8 and MutG360Δ9. Residues involved in cation-π interactions are shown in *stick* form.

The numbers of salt bridges detected in the tertiary structures of InuAMN8, MutG169Δ4, and MutG360Δ9 were 27, 26, and 27, respectively. The numbers of energetically significant cation-π interactions detected in the tertiary structures of InuAMN8, MutG169Δ4, and MutG360Δ9 were 6, 7, and 4, respectively. Notably, two cation-π interactions formed by amino acid residues F22 with R357 and F436 with R424 in InuAMN8 broke in MutG360Δ9 (Figure 5).

**FIGURE 6:**
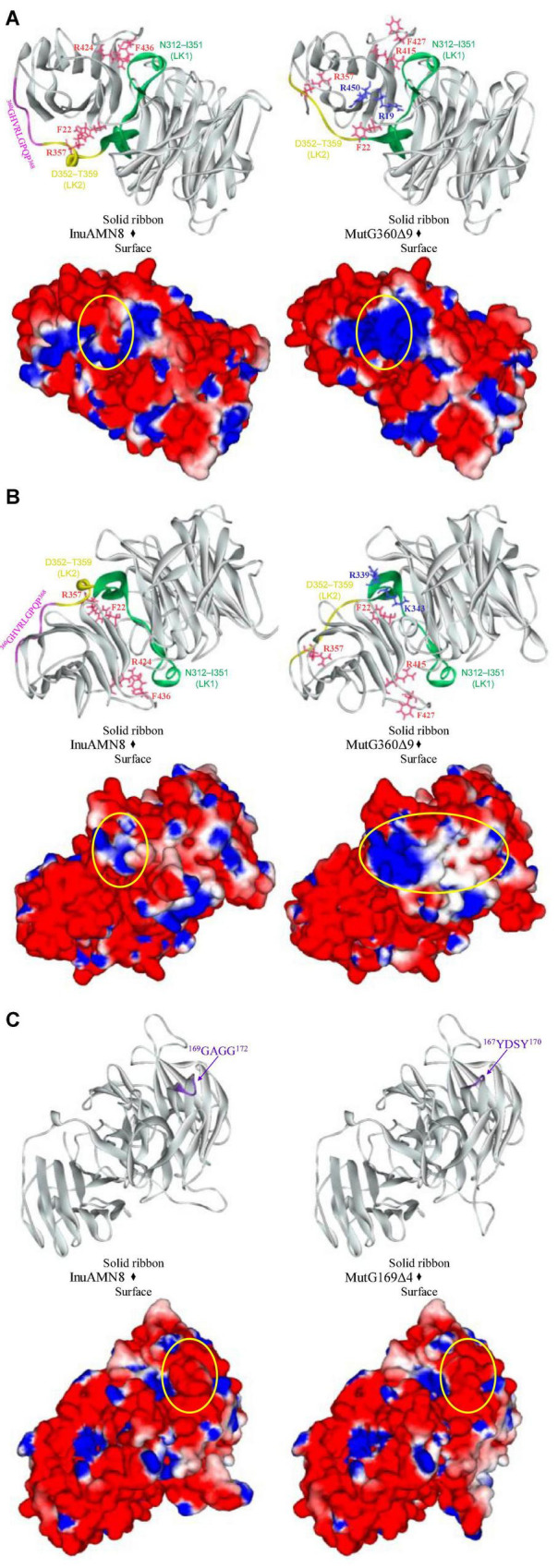
Comparisons of structures and charge distributions between wild-type InuAMN8 and its mutants. **(A)** Comparisons of structures and charge distributions between wild-type InuAMN8 and MutG360Δ9; **(B)** 180 degree rotation view of panel **(A)**; **(C)** comparisons of structures and charge distributions between wild-type InuAMN8 and MutG169Δ4. Positive charges are shown in *blue*, and negative charges are shown in *red*. The charge-changed surfaces are *circled*. Residues involved in charge-changed surfaces are shown in *blue* and *stick* form. Residues involved in cation-π interactions are shown in *peach* and *stick* form.

After removing ^360^GHVRLGPQP^368^, LK2 changed from a 3_10_-helix structure into a loop structure (Figure 6), and the residue R357 in LK2 was too far away from F22 to form a cation-π bond (Figure 5). The change of LK2 and break of the cation-π bond showed a Tamino domino effect on the Glyco_hydro_32C structure and the linking region: (1) the cation-π bond formed by F436 with R424 far from LK2 broke in the mutant MutG360Δ9 (Figure 5); (2) two basic amino acid residues R19 and R450 were exposed to the structural surface and changed the corresponding surface from negative electrostatic potential to positive electrostatic potential (Figure 6A); and (3) another two basic amino acid residues R339 and K343 in LK1 were also exposed to the structural surface and changed the corresponding surface from negative electrostatic potential to positive and neutral electrostatic potential (Figure 6B). However, the removal of ^169^GGAG^172^ did not expose any basic residue and changed surface potential (Figure 6C).

## Discussion

Loop structure plays an important role in the thermal performance of enzymes because it is usually located on the structural surface and has high flexibility that reduces the thermostability of enzymes (Vieille and Zeikus, 2001; Yu and Huang, 2014; Pucci and Rooman, 2017). To improve the thermostability of enzymes, loop structure is usually substituted, deleted, or shortened, such as in the studies for the effects of loops on the mannanase Man1312 from *Bacillus subtilis* B23 (Zhou H. Y. et al., 2016), the phospholipase D from *Streptomyces antibioticus* PLD (Damnjanovic et al., 2014), and the acylphosphatase from human muscle (Dagan et al., 2013). Loop structure also affected the thermal performance of exo-inulinases (Arjomand et al., 2016; He et al., 2022). Previously, Ω-loop 3 formed by ^74^YGSDVT^79^ of the exo-inulinase from *Aspergillus niger* 5012 was deleted and resulted in the optimum temperature decrease by 12°C and *t*_1/2_ decrease by 32 h at 60°C compared with the wild-type enzyme (Arjomand et al., 2016); Ω-loop 5 formed by ^137^EEDRK^141^ of the exo-inulinase InuAGN25 from *Sphingobacterium* sp. GN25 was deleted and resulted in the optimum temperature decrease by 10°C and *t*_1/2_ decrease by 31.7 min at 50°C compared with the wild-type enzyme (He et al., 2022). Both Ω-loop 3 and Ω-loop 5 are in the catalytic Glyco_hydro_32N domain. In this study, deletion of the Ω-loop formed by ^360^GHVRLGPQP^368^ in the linking region led similar thermal tolerance loss to previous studies for exo-inulinases; however, deletion of the Ω-loop formed by ^169^GGAG^172^ in the catalytic Glyco_hydro_32N domain did not lead thermal tolerance loss.

Thermal characteristics of enzymes are usually engineered through mutation for unconserved amino acid residue sites involved in intraprotein interactions (Vieille and Zeikus, 2001; Yu and Huang, 2014; Pucci and Rooman, 2017). Intraprotein interactions, including salt bridges and cation–π interactions, usually help to improve global and local rigidity of enzyme structure that hampers thermal denaturation of enzymes (Vieille and Zeikus, 2001; Pucci and Rooman, 2017; Siddiqui, 2017). A decrease in intraprotein interactions after loop deletion led to thermostability loss of exo-inulinases from *A. niger* 5012 and *Sphingobacterium* sp. GN25 (Arjomand et al., 2016; He et al., 2020, 2022). On the contrary, an increase in intraprotein interactions resulted in the thermostability enhancement of the exo-inulinase from *Sphingobacterium* sp. GN25 (He et al., 2020; Zhang et al., 2020). Therefore, the close numbers of salt bridges and cation-π interactions should be responsible for similar thermal characteristics of InuAMN8 and its mutant MutG169Δ4. In contrast, the loss of two cation-π interactions in MutG360Δ9 compared with InuAMN8 should play an important role for thermostability loss of the mutant.

In recent years, salt-tolerant enzymes have attracted much attention owing to their commercial and environmental values (Cao et al., 2021). The exo-inulinases from *Arthrobacter* strains HJ7 and MN8 and *Sphingomonas* sp. JB13 were observed to exhibit considerable salt tolerance, without a clear elucidation of structural characteristics and mechanisms regarding salt tolerance (Shen et al., 2015; Zhou et al., 2015a,b). Comparisons of biochemical and structural properties among InuAMN8 and its mutants MutG169Δ4 and MutG360Δ9 in this study revealed that salt tolerance showed a positive correlation with the negatively charged surface. Few studies also increased the negatively charged surfaces of enzymes through site-directed mutagenesis and caused the activity or stability enhancement of enzymes in salts (Warden et al., 2015) and vice versa (Li et al., 2019). To the best of our knowledge, most salt-tolerant enzymes have a structural characteristic of a larger negatively charged surface than their salt-sensitive counterparts, owing to that the negative electrostatic potential is capable of competing with salt ions for water molecules to form a stable hydration sphere that separates the protein molecules from each other in solution to avoid aggregating and collapsing (Warden et al., 2015; Cai et al., 2018; Cao et al., 2021).

Ethanol production using inulin is affected by ethanol tolerance of microbial strains and enzymes (Guo et al., 2013). However, alcohol-tolerant inulinases and related structural characteristics and mechanisms have not been reported. The molecular basis for enhanced tolerance of enzymes in organic solvents is very complex (Klibanov, 2001; Cui et al., 2020, 2021). Structural flexibility allows conformational mobility in increased intraprotein electrostatic interactions caused by organic solvents and then plays an important role in maintaining enzymatic activities (Klibanov, 2001; Li et al., 2018). The charged surface also contributes to the organic-solvent tolerance of enzymes. Some studies indicated that the activities or stabilities of enzymes improved, after that structural surfaces were substituted with charged electrostatic potential (Cui et al., 2020, 2021). For example, Cui et al. (2021) reported that the mutant M4 (I12R/Y49R/E65H/N98R/K122E/L124K) of *B. subtilis* lipase A was introduced charged amino acids to the structural surface and showed 2.1-fold activity increase in 30% (v/v) ethanol, in comparison with the wild-type enzyme. Therefore, the similar structural surface electrostatic potential resulted in similar alcohol-tolerant characteristics of InuAMN8 and its mutant MutG169Δ4, while the decrease in charged surface, especially negatively charged surface, in MutG360Δ9 compared with InuAMN8 accounted for the alcohol-tolerant loss of the mutant.

## Conclusion

The study revealed that the loop, which was constructed by ^360^GHVRLGPQP^368^ for linking domains of Glyco_hydro_32N and Glyco_hydro_32C of InuAMN8, affected thermo-halo-alcohol tolerance and structural properties. The loop was involved in the formation of two energetically significant cation-π bonds, which contributed to thermal properties of InuAMN8 at high temperatures. The loop was also involved in burying four basic amino acid residues with the ability to change the surface from negative electrostatic potential to positive and neutral electrostatic potential that caused detrimental effects on halo-alcohol tolerance. In the future, the loop in the linking region may be given attention for protein engineering in improving enzymatic properties in harsh environments.

## Data Availability Statement

The datasets presented in this study can be found in online repositories. The names of the repository/repositories and accession number(s) can be found in the article/Supplementary Material.

## Author Contributions

JZ and ZH: conceptualization. XC, RZ, and LH: data curation. XC and LH: formal analysis. JZ: funding acquisition, writing – review and editing, and methodology. ZH: project administration. XT, QW, and ZH: resources. RZ: writing – original draft. All authors contributed to the article and approved the submitted version.

## Conflict of Interest

The authors declare that the research was conducted in the absence of any commercial or financial relationships that could be construed as a potential conflict of interest.

## Publisher’s Note

All claims expressed in this article are solely those of the authors and do not necessarily represent those of their affiliated organizations, or those of the publisher, the editors and the reviewers. Any product that may be evaluated in this article, or claim that may be made by its manufacturer, is not guaranteed or endorsed by the publisher.
