# Supplementary material for: Deletion of the Loop Linking Two Domains of Exo-Inulinase InuAMN8 Diminished the Enzymatic Thermo-Halo-Alcohol Tolerance
Source: Front Microbiol. 2022 Jun 23;13:924447. doi: 10.3389/fmicb.2022.924447 (PMC9260423; doi:10.3389/fmicb.2022.924447)
Supplement: Supplementary file 1 [file Data_Sheet_1.docx]

Deletion of the Loop Linking Two Domains of Exo-Inulinase InuAMN8 Diminished the Enzymatic Thermo-Halo-Alcohol Tolerance

Xiaolong Cen ^1,2,3,4^, Rui Zhang ^1,2,3,4^, Limei He ^1,2,3,4^, Xianghua Tang ^1,2,3,4^, Qian Wu ^1,2,3,4^, Junpei Zhou ^1,2,3,4^*, Zunxi Huang ^1,2,3,4^*

^1^ Engineering Research Center of Sustainable Development and Utilization of Biomass Energy, Ministry of Education, Yunnan Normal University, Kunming 650500, Yunnan, PR China

^2^ College of Life Sciences, Yunnan Normal University, Kunming 650500, Yunnan, PR China

^3^ Yunnan key Laboratory of Biomass Energy and Enviromnental Biotechnology, Yunnan Normal University, Kunming 650500, Yunnan, PR China

^4^ Key Laboratory of Yunnan Provincial Education Department for Plateau Characteristic Food Enzymes, Yunnan Normal University, Kunming 650500, Yunnan, PR China

*** Correspondence:**Junpei Zhou
junpeizhou@ynnu.edu.cn; junpeizhou@126.com

*** Correspondence:**Zunxi Huang
huangzunxi@163.com

**Supplementary Figure 1.** Ramachandran Plot of the homology models of InuAMN8 and its mutants.

**Supplementary Figure 2.** VERIFY3D results of the homology models of InuAMN8 and its mutants.
